# *LRRK2*, *GBA* and their interaction in the regulation of autophagy: implications on therapeutics in Parkinson's disease

**DOI:** 10.1186/s40035-022-00281-6

**Published:** 2022-01-31

**Authors:** Shirley Yin-Yu Pang, Rachel Cheuk Nam Lo, Philip Wing-Lok Ho, Hui-Fang Liu, Eunice Eun Seo Chang, Chi-Ting Leung, Yasine Malki, Zoe Yuen-Kiu Choi, Wing Yan Wong, Michelle Hiu-Wai Kung, David Boyer Ramsden, Shu-Leong Ho

**Affiliations:** 1 Division of Neurology, Department of Medicine, Queen Mary Hospital, University of Hong Kong, Hong Kong, China; 2 School of Biomedical Sciences, Li Ka Shing Faculty of Medicine, University of Hong Kong, Hong Kong, China; 3 Institute of Metabolism and Systems Research, University of Birmingham, Birmingham, UK

**Keywords:** Parkinson’s disease, Interaction, LRRK2, GBA, GCase, Mutation, Autophagy, α-Synuclein

## Abstract

Mutations in leucine-rich repeat kinase 2 (*LRRK2*) and glucocerebrosidase (*GBA*) represent two most common genetic causes of Parkinson’s disease (PD). Both genes are important in the autophagic-lysosomal pathway (ALP), defects of which are associated with α-synuclein (α-syn) accumulation. LRRK2 regulates macroautophagy *via* activation of the mitogen activated protein kinase/extracellular signal regulated protein kinase (MAPK/ERK) kinase (MEK) and the calcium-dependent adenosine monophosphate (AMP)-activated protein kinase (AMPK) pathways. Phosphorylation of Rab GTPases by LRRK2 regulates lysosomal homeostasis and endosomal trafficking. Mutant LRRK2 impairs chaperone-mediated autophagy, resulting in α-syn binding and oligomerization on lysosomal membranes. Mutations in *GBA* reduce glucocerebrosidase (GCase) activity, leading to glucosylceramide accumulation, α-syn aggregation and broad autophagic abnormalities. *LRRK2* and *GBA* influence each other: GCase activity is reduced in *LRRK2* mutant cells, and LRRK2 kinase inhibition can alter GCase activity in *GBA* mutant cells. Clinically, *LRRK2* G2019S mutation seems to modify the effects of *GBA* mutation, resulting in milder symptoms than those resulting from *GBA* mutation alone. However, dual mutation carriers have an increased risk of PD and earlier age of onset compared with single mutation carriers, suggesting an additive deleterious effect on the initiation of PD pathogenic processes. Crosstalk between *LRRK2* and *GBA* in PD exists, but its exact mechanism is unclear. Drugs that inhibit LRRK2 kinase or activate GCase are showing efficacy in pre-clinical models. Since LRRK2 kinase and GCase activities are also altered in idiopathic PD (iPD), it remains to be seen if these drugs will be useful in disease modification of iPD.

## Background

Autophagy is a degradation process to remove proteins and dysfunctional organelles from cells to prevent subsequent toxicity and cell death. There are three forms of autophagy: (1) macroautophagy, which involves sequestration of portions of the cytosol into double-membrane vesicles or autophagic vacuoles (AV) that then fuse with lysosomes [1]; (2) chaperone-mediated autophagy (CMA), which involves the direct transport of cytosolic soluble proteins across the lysosomal membrane in a selective fashion [2]; and (3) microautophagy, which involves sequestration of cytosolic contents directly by lysosomes through membrane invagination [3]. Parkinson’s disease (PD), the second most common neurodegenerative disease after Alzheimer’s disease, is characterized pathologically by loss of dopaminergic neurons in the substantia nigra pars compacta and intraneuronal inclusions called Lewy bodies (LB) that consist of aggregated α-synuclein (α-syn) [4, 5]. In postmortem PD brains, reduction of lysosomal markers is apparent in nigral neurons that contain α-syn inclusions [6]. Furthermore, lysosomal depletion has been shown to precede dopaminergic cell death in a PD mouse model [7]. Since α-syn is degraded in lysosomes, impairment of the autophagic-lysosomal pathway (ALP) could lead to impaired α-syn clearance. Aggregation of α-syn into toxic oligomers then further aggravates the impairment in autophagic and lysosomal functions, forming a vicious cycle [6–8].

Genetic studies have indicated that malfunctioning degradation pathways contribute to the pathogenesis of PD. Only 5%–10% of PD patients have familial forms of the disease [8], and PD has traditionally been considered as a largely sporadic disease. However, advancements in our understanding of the genetic basis of PD suggest that genetic factors can cause or increase the susceptibility to PD to a much larger extent than previously thought: the heritability of PD has been estimated to be at least 27% and up to 60% in large genome-wide association studies (GWAS) (reviewed in [9]). Genes and genetic loci identified in familial and sporadic PD are strongly enriched for autosomal/lysosomal functions: among the 24 loci identified by GWAS to be associated with PD [10], at least 11 genes are implicated in the ALP [9, 11]. In particular, mutations in *LRRK2* (encoding leucine-rich repeat kinase 2, LRRK2) and *GBA* (encoding glucocerebrosidase, GCase) are now recognized as two most common genetic causes of PD worldwide [9]. Recent evidence in experimental models of PD suggests that *LRRK2* and *GBA* are closely related to the regulation of ALP [12–14]. Importantly, enzymatic activities of LRRK2 and GCase have also been shown to be altered in idiopathic PD (iPD) [15–17].

Dopaminergic neuronal loss in late-onset PD starts 20–30 years before first motor symptoms of rest tremor, rigidity and bradykinesia appear, by which time there is already 50% striatal dopamine (DA) reduction [18]. There is a long prodromal or pre-motor period during which non-motor symptoms such as hyposmia and rapid eye movement sleep behavior disorder (RBD) already start to emerge [19]. It is hypothesized that during different stages of the disease, α-syn aggregates into oligomeric species, which then seed further aggregation and spread within the nervous system in a prion-like fashion [20, 21]. The long prodromal period represents a window of opportunity to modify disease progression. Understanding the roles *LRRK2* and *GBA* play in autophagy and α-syn aggregation will help elucidate the pathogenesis of PD and formulate rational therapeutic strategies.

## LRRK2 and autophagy

### LRRK2 mutations in PD

Mutations in *LRRK2*, located in the *PARK8* locus, are the most common mutations in familial autosomal-dominant PD [22, 23], and *LRRK2* polymorphisms are associated with increased PD risk in GWAS [24], suggesting a role of *LRRK2* in both sporadic and familial PD. Pleomorphic pathology including tauopathy or pure nigral degeneration has been reported in rare cases. Nevertheless, most *LRRK2*-PD cases have clinical and pathological features indistinguishable from iPD with late-onset disease, dopaminergic neuron degeneration in the substantia nigra and intracytoplasmic LB aggregates with positive staining for α-syn [23].

The LRRK2 protein is ubiquitously expressed, with highest levels in kidney, lung and brain (reviewed in [25]). It consists of multiple domains: armadillo repeats, ankyrin repeats, leucine-rich repeats, Ras of complex (Roc) with GTPase activity, C-terminal of Roc (COR), kinase, and WD40 domains [26]. The two most common mutations, G2019S located in the kinase domain of *LRRK2* and R1441C/G/H located in the GTPase domain, account for up to 10% and 2.5% of sporadic iPD cases, respectively [27]. Structural analyses of LRRK2 showed that the kinase and GTPase domains are in close proximity and can influence each other [28]. All known pathogenic *LRRK2* mutations, including G2019S and R1441C/G/H, can lead to increased kinase activity [29–32], suggesting that the increased phosphorylation of LRRK2 kinase substrates may result in toxicity to dopaminergic neurons.

### LRRK2 and regulation of macroautophagy

Under normal conditions, autophagy occurs at a basal level to maintain homeostasis. When cells are under stress, autophagy promotes cell survival against apoptosis, but in some settings it can also cause cell death [33]. There is evidence that *LRRK2* plays a role in the regulation of macroautophagy. Accumulation of AV, shortened neurite length and reduced neuronal survival have been noted in rat neurons overexpressing PD-associated *LRRK2* mutant proteins in a LRRK2 kinase-dependent manner [34]. These abnormalities are not seen in cells that overexpress wild-type (WT) *LRRK2* or the kinase-dead *LRRK2* K1906M mutant, suggesting that the increased LRRK2 kinase activity is responsible for the abnormalities observed. Accumulation of AV has also been identified in dopaminergic neurons in the substantia nigra of iPD patients [35]. The increased amount of AV may be due to the increased induction of macroautophagy, reduced clearance of autophagosomes, or both. Fibroblasts from PD patients with *LRRK2* G2019S mutation have increased basal macroautophagy as evidenced by increased numbers of autophagosomes and autolysosomes, increased protein degradation, and increased cell death [36]. Induction of macroautophagy by rapamycin in human neuroblastoma cells overexpressing *LRRK2* G2019S exacerbates autophagosome accumulation and neurite shortening, confirming that excessive macroautophagy induction can cause stress in susceptible cells [37]. Furthermore, these abnormalities can be reversed by inhibition of the mitogen activated protein kinase/extracellular signal regulated protein kinase (MAPK/ERK) kinase (MEK), suggesting that the increased LRRK2 kinase activity leads to activation of the MEK/ERK pathway, excessive macroautophagic induction and cell death [36, 37]. Another pathway implicated in autophagosome accumulation in *LRRK2* mutant cells is the Ca^2+^-dependent activation of the CaMKK/adenosine monophosphate (AMP)-activated protein kinase (AMPK) pathway, which can be blocked by calcium chelation or by treatment with a specific antagonist of the Ca^2+^-mobilizing messenger nicotinic acid adenine dinucleotide phosphate (NAADP), suggesting that NAADP receptors may be targets for regulation by *LRRK2* [38].

Cellular stress, such as starvation, can induce macroautophagy by inhibiting the mammalian target of rapamycin (mTOR) [39]. Interestingly, while rapamycin as an inhibitor of mTOR induces macroautophagy similar to that occurring in cells overexpressing *LRRK2* G2019S, the macroautophagy inhibitor 3-methyladenine reverses autophagosome accumulation induced by rapamycin but not by *LRRK2* mutation. This suggests a mechanistic difference between the mTOR- and *LRRK2*-mediated macroautophagy induction [38]. When cells are further stressed with a proteasome inhibitor, cell death is markedly increased in *LRRK2* mutant cells, which can be rescued by rapamycin that increases autophagic flux through the mTOR pathway.

Collectively, these studies show that the mutant LRRK2 protein with increased kinase activity causes excessive induction of basal macroautophagy, AV accumulation and cell death. Conversely, following proteasomal inhibition, cells with mutant LRRK2 show reduced degradative capacity and survival, which can be rescued by macroautophagy induction via the mTOR pathway. These observations suggest that *LRRK2* plays an important regulatory role in autophagic balance under different cellular conditions, disturbance of which may lead to reduced cell survival.

### LRRK2 and lysosomal function

In addition to autophagosome induction, *LRRK2* mutation also compromises the maturation of autophagosomes into autolysosomes as shown by reduced co-localization of light chain 3 (LC3; an autophagosome marker) with lysosome-associated membrane protein 1 (LAMP1) [40]. Furthermore, lysosomes are abnormal with increased alkalinization and reduced protein degradation in a *LRRK2* R1441C transgenic mouse model and in SH-SY5Y cells overexpressing *LRRK2* G2019S, highlighting the role of *LRRK2* in lysosomal biology [41, 42]. Lysosomal dysfunction in *LRRK2* mutant cells is associated with increased detergent-insoluble α-syn, accumulation of phosphorylated α-syn at serine 129 (pS129-α-syn), and increased neuronal release of α-syn, all of which can be reversed by pharmacologic inhibition of LRRK2 kinase [14, 42, 43]. Interestingly, in WT cells, the same phenotype of abnormal lysosomal morphology and increased insoluble α-syn can be induced by treatment with lysosomal inhibitors, indicating that lysosomal inhibition can increase insoluble α-syn in WT cells similarly to that seen in *LRRK2* mutant cells, thereby confirming the importance of functional lysosomes in α-syn degradation [42].

LRRK2 may regulate lysosomal function through its kinase activity on a subset of Rab GTPases, which have been shown to be bona fide substrates of LRRK2 [31]. Upon lysosomal stress, LRRK2 is recruited by Rab7L1 (also called Rab29) from the cytoplasm onto enlarged lysosomes [44]. Furthermore, Rab8a and Rab10 accumulate in LRRK2-positive enlarged lysosomes in a LRRK2 kinase-dependent manner. Collectively, the sequential recruitment of Rab7L1, LRRK2, phosphorylated Rab8a and Rab10 onto lysosomes under stress suppresses lysosomal enlargement and promotes release of lysosomal content, illustrating the role of the Rab7L1-LRRK2 pathway in lysosome homeostasis. LRRK2 and Rab7L1 are also involved in retromer function which is required for retrograde transport of selective cargos between endosome and Golgi [45]. Disruption of the retromer function by *LRRK2* mutation leads to impairment in recruitment of lysosomal hydrolases, and lysosomal deficits. Another LRRK2 substrate, Rab35, is increased and colocalized with α-syn on enlarged endosomes in transgenic mice overexpressing α-syn [46]. Treatment with LRRK2 kinase inhibitor reduces Rab35 levels and its co-localization with α-syn, normalizes the size of enlarged endosomes and increases co-localization of α-syn with cathepsin, indicating increased trafficking of α-syn to lysosome for degradation. Collectively, this series of studies suggests that LRRK2 regulates lysosomal function through its kinase activity on a subset of Rab GTPases.

### LRRK2 and CMA

There is evidence that CMA is perturbed in PD: lysosome-associated membrane protein 2A (LAMP2A), which multimerizes to form a translocation complex on lysosomal membranes essential for CMA, is reduced in the substantia nigra of postmortem PD brain samples [47]. Notably, α-syn and LRRK2 are both substrates of CMA and their paths may converge in the lysosome [48, 49]. Mutant LRRK2 binds to lysosomal membranes less efficiently than WT LRRK2, but once bound, its binding to LAMP2A is more stable and it seems to prevent LAMP2A multimerization to form the translocation complex, leading to impaired degradation of other CMA substrates including α-syn [49]. Furthermore, rather than competing with α-syn for binding to LAMP2A, mutant LRRK2 actually enhances binding of monomeric α-syn to lysosomal membranes. Since LAMP2A multimerization is blocked by mutant LRRK2, α-syn bound to lysosomal membrane would not be translocated into the lysosome, resulting in a marked increase of the formation of α-syn oligomers at the surface of lysosomes. Based on observations in induced pluripotent stem cell (iPSC)-derived DA neurons from PD patients with *LRRK2* mutation, alterations in CMA appear to be an early event, detectable before impaired macroautophagy and overt neurodegeneration [40, 49]. In light of these findings, CMA activation has been explored as a therapeutic strategy. Our study using a *LRRK2* R1441G-knockin mouse model of PD has shown an age-dependent accumulation of oligomeric α-syn, increased LAMP2A levels, and impaired CMA and lysosomal activity [50]. Treatment of cells with a CMA activator increases lysosomal activity and reduces intra- and extra-cellular α-syn oligomers in primary cortical neurons back to the levels comparable to WT, suggesting that activation of CMA may be a viable therapeutic strategy to reduce α-syn accumulation and release.

**Fig. 1:**
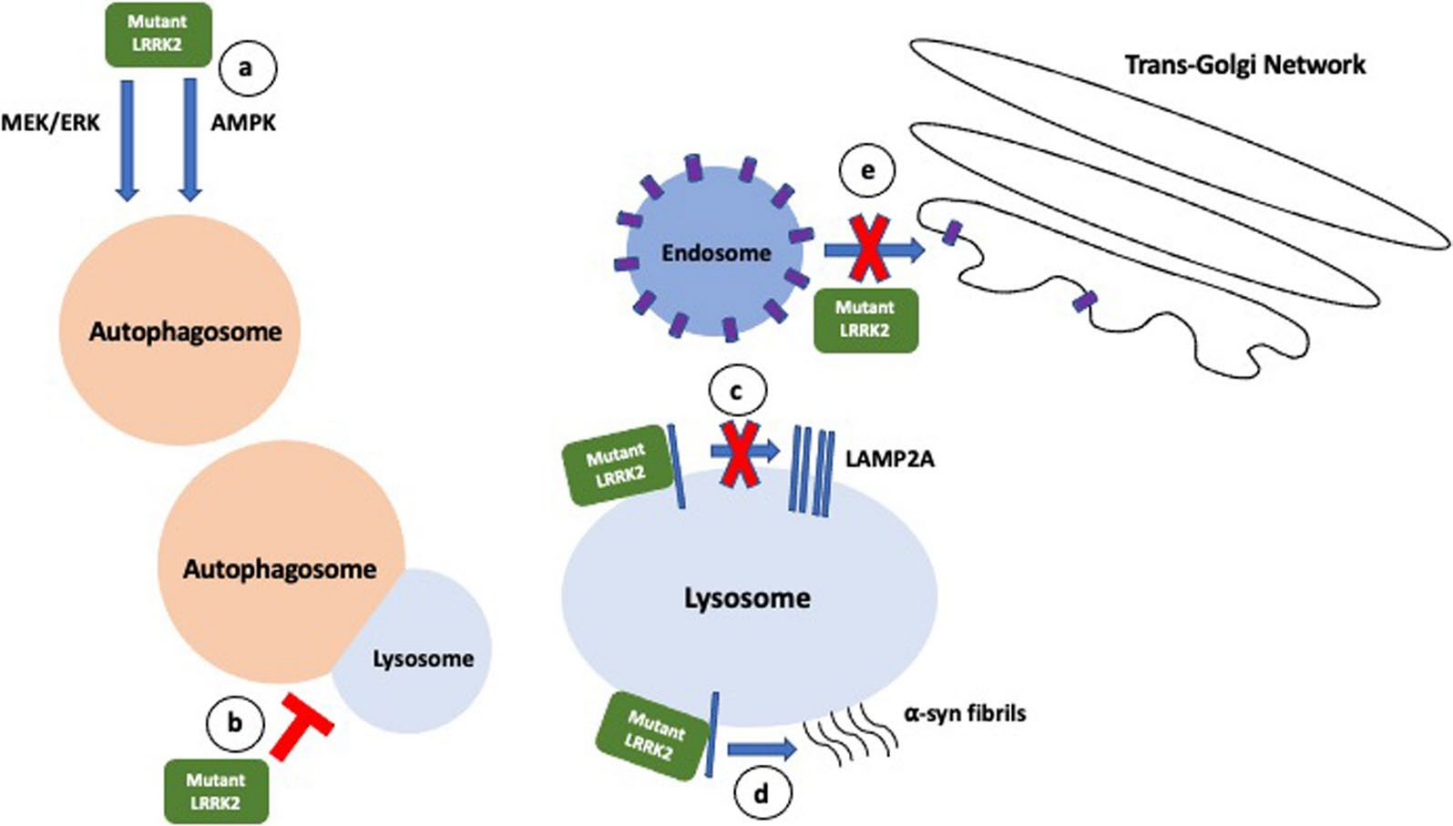
Defects in autophagy associated with LRRK2 mutations. **a** Mutant LRRK2 induces autophagosome formation by activating the MEK/ERK pathway and the Ca^2+^-dependent AMPK pathway. **b** Fusion of autophagosome with lysosome is blocked, exacerbating autophagosome accumulation. **c** Mutant LRRK2 binds to LAMP2A and prevents its multimerization to form the translocation complex required for transport of CMA substrates into lysosome for degradation. **d** Mutant LRRK2 promotes binding of α-syn onto lysosomal membranes where they form oligomers. **e** Impaired protein trafficking from endosome to trans-Golgi network due to retromer dysfunction leads to hydrolase deficiencies in lysosome

In summary, the PD-associated pathogenic *LRRK2* mutations increase phosphorylation of LRRK2 kinase substrates *in vivo* [31] and are associated with: (1) alterations in the regulation of macroautophagy under different cellular conditions, (2) impaired lysosomal function with abnormal lysosomal morphology and increased alkalinization, (3) altered endolysosomal trafficking mediated by increased phosphorylation of a subset of Rab GTPases, and (4) impaired CMA by enhanced binding to LAMP2A and blockage of degradation of other CMA substrates including α-syn. These abnormalities likely contribute to α-syn accumulation and oligomerization in *LRRK2*-PD (Fig. 1).

## GBA and autophagy

### *GBA* mutations in PD

*GBA* encodes the lysosomal enzyme GCase, which cleaves the glucose moiety from glucosylceramide (GlcCer). Homozygous mutations of *GBA*, resulting in GCase enzymatic deficiency, cause Gaucher disease (GD) in which affected cells are engorged with abnormal lysosomes containing the GCase substrate, GlcCer (reviewed in [51–53]). *GBA* is located on chromosome 1q21. At least 495 mutations, including missense, frameshift, splice-site mutations and null alleles resulting from recombination with the homologous *GBA* pseudogene have been described in GD [53]. The prevalence of different *GBA* mutations varies with ethnicity. N370S is the most common mutation among Ashkenazi Jews, while L444P is more prevalent in Asians and Caucasians with non-Ashkenazi Jew ancestry [51, 52]. The earliest clues of *GBA* involvement in PD came from observations that GD patients and their relatives had increased incidence of PD compared with the general population [54, 55]. Heterozygous *GBA* mutation carriers have a 10%–30% probability of developing PD at the age of 80 (a 20-fold rise compared to non-mutation carriers) (reviewed in [56]). Moreover, *GBA* mutations occur in 5%–10% of PD patients, making *GBA* mutations the most significant genetic risk factor for PD [55, 57]. The most common *GBA* mutations in PD patients worldwide are N370S and L444P [52]. The pathogenicity of *GBA* mutations in PD is thought to be related to reduced GCase activity (i.e. loss-of-function) as severe *GBA* mutations appear to be correlated with a higher risk of PD development and significantly worse motor and non-motor symptoms compared with mild mutations [58, 59]. Patients with *GBA*-associated PD (*GBA*-PD) have similar motor symptoms as iPD, but may have earlier age of onset and increased prevalence of cognitive impairment [60, 61]. *GBA*-PD is also shown to have similar brain pathology in terms of Lewy-type synucleinopathy to non-*GBA* PD subjects [61]. GCase activity has been found to be reduced in the caudate and substantia nigra of iPD patients [17, 62], suggesting that GCase dysfunction is a common pathogenic mechanism in iPD. However, in addition to GCase enzymatic deficiency, it is likely that other pathogenic mechanisms are also involved. Not all GD patients, even those with severe *GBA* mutations, develop PD and some variants, notably E326K and T369M, confer increased risk of PD but do not cause GD [56, 63]. Although no mechanisms have been established for the pathogenicity of the latter variants, a gain-of-function mechanism is possible where mutated and misfolded GCase protein accumulates in the endoplasmic reticulum (ER), leading to ER stress, ER-associated degradation and cell death (reviewed in [53, 56]). Moreover, GCase has been shown to be present in LBs [64]. Overall, mutations in *GBA* represent a genetic risk factor for PD as penetrance is incomplete, and both loss-of-function and gain-of-function mechanisms have been proposed. Regardless of the degree of GCase deficiency, *GBA*-PD is characterized by increased α-syn aggregation, the mechanisms of which will be discussed below.

### GBA and lysosomal function

GCase is synthesized in the ER and transported by lysosomal integral membrane protein 2 (LIMP2) to the lysosome. Upon reaching the lysosomal lumen, GCase becomes active and hydrolyzes GlcCer to ceramide and glucose (reviewed in [11, 56]). The link between GCase deficiency and synucleinopathy was first reported in neuropathological studies of GD patients with parkinsonism, which revealed the presence of LBs and α-syn aggregation in the hippocampus [65, 66]. Since GCase is a lysosomal enzyme, GCase deficiency may perceivably alter lysosomal function, leading to defective protein degradation and synucleinopathy. Indeed, knockdown of *GBA* in primary cortical neurons results in reduced GCase activity, increased accumulation of its substrate GlcCer, reduced rate of lysosomal proteolysis, accumulation of enlarged lysosomes, and increased α-syn without increasing its mRNA (suggesting that the increased α-syn is due to reduced degradation) [67]. Neuroblastoma cells with *GBA* knockout have increased accumulation of lysosomal substrates p62 and polyubiquinated proteins, increased Lysotracker staining indicative of reduced breakdown of acidic organelles, increased abnormal accumulation of enlarged autophagic vesicles and increased insoluble α-syn as well as α-syn release, further illustrating the critical role of GCase activity in maintaining normal lysosomal function and α-syn homeostasis [68]. DA neurons derived from iPSCs of *GBA*-PD patients carrying heterozygous *GBA* mutations show reduced GCase activity and increased accumulation of GlcCer and α-syn compared with control DA neurons [69]. Defects in ALP are evident due to the following alterations: (1) increased LAMP1-positive puncta suggesting accumulation of lysosomes, (2) reduced activity of other lysosomal enzymes, (3) increased LC3-positive vesicles, and (4) reduced co-localization between LC3 and LAMP1 vesicles, indicating impaired autophagosome-lysosome fusion. Importantly, these abnormalities are rescued by correction of the *GBA* mutations. Furthermore, control neurons treated with a GCase inhibitor show increased α-syn levels similar to *GBA*-mutant neurons. Collectively, these studies suggest that GCase deficiency causes numerous abnormalities in the ALP: accumulation of lysosomes, reduced activity of lysosomal enzymes, autophagosome accumulation with impaired maturation, accumulation of the GCase substrate GlcCer, and increased insoluble α-syn and α-syn release.

Lysosome biogenesis and recycling are important for cellular homeostasis. Lysosomal proteins are transported to the lysosome *via* the endosomal system, where early endosomes mature to late endosomes, which then fuse with the lysosome, delivering their cargo [70]. There is evidence that GCase deficiency impairs lysosome biogenesis *via* autophagic lysosome reformation [71]. Normally, after degradation of autolysosomal products, mTOR is activated to terminate autophagy and phosphorylates its substrate p70S6Kinase (phopho-S6K). This leads to formation of proto-lysosomal tubules in the autolysosomes; these tubules are ultimately excluded from autolysosomes to mature into functional lysosomes *via* the endosomal system [70]. Mouse embryonic fibroblasts with *GBA* knockout or heterozygous *GBA* mutation have reduced levels of phopho-S6K, which can be reversed by recombinant GCase enzyme replacement, confirming the direct relationship between loss of GCase activity and loss of mTOR activity [71]. Furthermore, these cells exhibit increased levels of Rab7 (a marker of late endosomes) and increased co-localization of Rab7 with the lysosomal enzyme cathepsin D, suggesting slower dissociation of proto-lysosomes from autolysosomes and slower lysosome maturation and recycling. Over time, with repeated cycles of autophagy followed by autophagic lysosome reformation, this would conceivably result in fewer functional lysosomes, contributing to lysosomal dysfunction in *GBA*-mutant cells.

### GCase and α-syn: a bi-directional loop?

Knockdown of GCase in neurons causes accumulation of GlcCer, reduced rate of proteolysis, accumulation and enlargement of lysosomal compartment, and increased levels of soluble monomeric, oligomeric and insoluble α-syn [67]. When these cells over-express WT or A53T α-syn, there is a significant decline in cell viability compared with cells with normal GCase; interestingly, cell viability is not reduced if the cells with GCase knockdown over-express an artificially generated fibrillation-incompetent α-syn mutant, suggesting that GCase knockdown promotes accumulation and neurotoxicity of α-syn through polymerization-dependent mechanisms. Intriguingly, the effect of lysosomal dysfunction in GCase deficiency seems to preferentially affect α-syn, since the levels of other aggregation-prone proteins such as tau and huntingtin are not increased in GCase-knockdown cells. Furthermore, treatment with the lysosomal inhibitor leupeptin results in increased total insoluble proteins but does not increase levels of soluble oligomeric α-syn, while knockdown of GCase results in increased levels of soluble oligomeric α-syn but not total insoluble proteins. This suggests that GCase deficiency preferentially affects the solubility of α-syn and that this effect is due to alteration of the GlcCer pathway rather than a result of general lysosomal inhibition. In vitro data have shown that increasing the concentrations of GlcCer can stabilize the formation of a soluble assembly-competent intermediate α-syn species and promote α-syn fibril formation, thus offering a potential mechanism by which GlcCer accumulation in GCase deficiency may promote synucleinopathy. These observations are corroborated in GD mouse brain showing reduced GCase activity, accumulation of GlcCer, and degeneration of neurons in substantia nigra and cortex, with increased soluble oligomeric and insoluble α-syn [67].

Decreased GCase activity has also been noted in postmortem brain samples of iPD patients; furthermore, the decrease in GCase activity in the substantia nigra of PD patients correlates with increased α-syn levels [16, 17, 65, 72]. In SH-SY5Y cells, over-expression of α-syn reduces GCase activity in a dose-dependent manner, suggesting that α-syn accumulation can lead to reduced activity of WT GCase in cells with no *GBA* mutations. A study on postmortem brain samples of early-stage iPD patients has shown reduced GCase activity selectively in brain regions that accumulate α-syn [16]. Furthermore, even though there is no change in constituent lysosomal membrane proteins (indicating no overt loss or accumulation of lysosomes), there is evidence of impaired CMA with reduced LAMP2A levels that correlates with increased α-syn and reduced GCase activity, suggesting that these are early events in the clinical course of PD.

α-Syn accumulation likely causes reduced GCase activity by interfering with the trafficking of GCase [66]. Normally, GCase binds the lysosomal transporter LIMP2 in the ER and is transported *via* the Golgi apparatus to lysosomes. In neurons overexpressing α-syn, LIMP2 fails to bind GCase and there is increased trapping of GCase in the ER, with concomitant reduction of GCase activity in lysosomes [17, 67]. The mechanism underlying this is unclear since LIMP2 does not appear to bind α-syn. Interestingly, this effect is not observed if mutant α-syn lacking amino acids 71–82 (i.e. fibrillation-incompetent α-syn) is overexpressed, again suggesting that the impairment in ER-to-lysosome trafficking of GCase is dependent on polymerization of α-syn [67]. Retention of GCase in ER may induce ER stress, as shown by activation of the unfolded protein response in DA neurons differentiated from iPSC of *GBA*-PD patients, together with numerous defects in autophagy: increased autophagosomes, impaired lysosomal protein degradation, increased number of lysosomes and increased α-syn release [73].

**Fig. 2:**
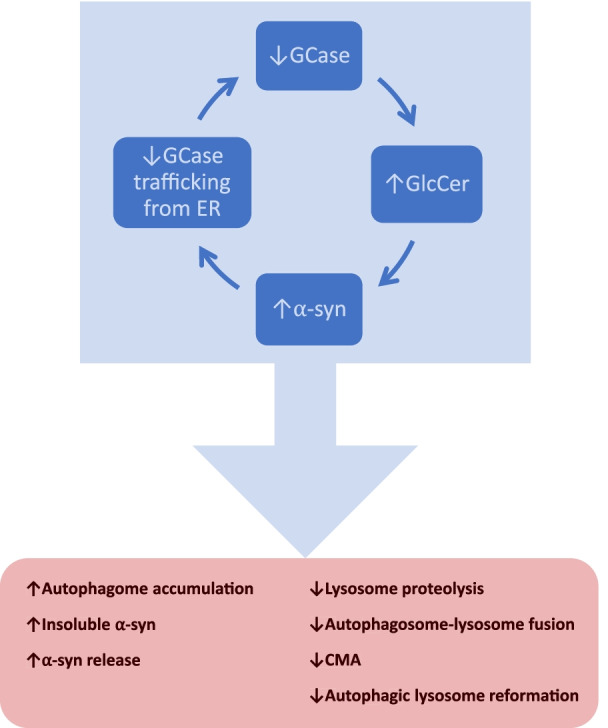
Vicious cycle of GCase deficiency and α-syn accumulation is associated with multiple defects in autophagic-lysosomal pathway. Reduced GCase activity leads to accumulation of its substrate GlcCer, which acts as a scaffold for α-syn fibril formation. α-Syn aggregation impairs the transport of GCase from ER to lysosome, further reducing lysosomal GCase activity

In summary, *GBA* mutations likely increase PD risk by the following proposed mechanisms: (1) gain-of-function mechanism where mutant and misfolded GCase accumulates in ER, causing ER stress, (2) loss-of-function mechanism where GCase deficiency causes accumulation of its substrate GlcCer, which stabilizes and promotes α-syn aggregation, and (3) a bi-directional loop where oligomeric α-syn interferes with GCase trafficking, further exacerbating GCase deficiency, leading to more α-syn aggregation. These changes are associated with broad abnormalities in the ALP (Fig. 2).

## Crosstalk between *LRRK2* and *GBA*

Both *LRRK2* and *GBA* play critical roles in the ALP. Mutations in either gene cause similar dysfunction in macroautophagy, lysosomal biology and CMA, resulting in the aggregation and propagation of α-syn, raising the possibility that *LRRK2* and *GBA* mutations may contribute to PD pathogenesis through a common biological pathway. Dissecting how the two genes interact and regulate autophagy may identify potential therapeutic targets for disease modification in PD.

Crosstalk between *LRRK2* and *GBA* can been seen in DA neurons with *LRRK2* R1441C, R1441G or G2019S mutation, which show reduced GCase activity [14]. GCase activity can be restored by treatment with LRRK2 kinase inhibitor, indicating that *LRRK2* mutations reduce GCase activity in a kinase-dependent manner. Rab10 is a key mediator of GCase activity and is regulated by *LRRK2*: phosphorylation of Rab10 by LRRK2 reduces GCase activity. Notably, treatment with LRRK2 kinase inhibitor also increases GCase activity in DA neurons derived from healthy controls and from PD patients with heterozygous *GBA* mutation, suggesting that LRRK2 kinase regulates GCase activity irrespective of the mutation status or the disease state [14]. The increase in GCase activity after treatment with LRRK2 kinase inhibitor in *LRRK2*-mutant and in *GBA*-mutant neurons is accompanied by the reduction of pS129-α-syn, the predominant form of α-syn found in LBs [5, 74].

Neurons with heterozygous-null *GBA* mutation with apparent normal LRRK2 kinase activity show broad lysosomal impairment and increased α-syn accumulation and release [12]. Despite having normal intrinsic LRRK2 kinase activity, treatment of these *GBA*-mutant neurons with LRRK2 kinase inhibitor results in near complete rescue of lysosomal deficits, supporting a functional link between the two proteins in the regulation of lysosomal function [12]. Similarly, *GBA*-mutant astrocytes do not have elevated intrinsic LRRK2 kinase activity but show impaired basal and evoked cytokine production, which can be reversed with LRRK2 kinase inhibitor, indicating the possibility of a broader effect on immune response exerted by *GBA–LRRK2* crosstalk [13]. Collectively, these studies indicate that *LRRK2* and *GBA* influence each other in the regulation of lysosomal function and that LRRK2 kinase inhibitor may be a potential treatment strategy to correct defects in lysosome and cytokine response in not only *LRRK2*-PD but also *GBA*-PD or perhaps even iPD.

## Dual *LRRK2-GBA* mutations in PD patients

Since *LRRK2* and *GBA* mutations are two most common genetic causes of PD, patients with mutations in either gene or in both genes are increasingly reported, with an opportunity to study the effects of these mutations on phenotype. Two studies, which include 503 *LRRK2*-PD patients, the majority (89%) being G2019S mutation carriers, show that the motor phenotypes of *LRRK2*-PD are generally indistinguishable from iPD [27, 75]. Studies of non-motor features in 485 *LRRK2*-PD patients (480 or 99% being G2019S carriers) show conflicting results. Some report higher rates of depression in *LRRK2* G2019S patients [76], while others show no significant difference in depression and anxiety in *LRRK2* G2019S carriers compared with non-carriers [77, 78]. Cognitive function is similar in *LRRK2* G2019S carriers and non-carriers in some studies [79, 80], while others show better cognitive function with lower rates of dementia in *LRRK2* G2019S carriers [27, 81]. *GBA* carriers have been observed to have a more rapid motor decline and a higher burden of nonmotor features, specifically dementia, depression and anxiety, than iPD patients [82–87]. In particular, severe *GBA* mutations (e.g. L444P) are associated with a higher risk of PD, earlier age of onset, more rapid progression and worse cognitive functions than mild mutations (e.g. N370S) [59, 88, 89]. The age of onset is comparable between *LRRK2* carriers and iPD, but is significantly earlier in *GBA*-PD [77, 78, 90, 91]. In PD patients with *GBA* mutations, the age of onset in those with severe *GBA* mutations is up to 8 years earlier than patients with iPD, while mild *GBA* mutation carriers have similar age of onset as iPD patients [91, 92]. Overall, *GBA*-PD patients seem to have worse motor and non-motor symptoms than iPD while *LRRK2*-PD patients are more similar to iPD. However, current evidence is not sufficient to distinguish *GBA-* or *LRRK2-*PD from iPD by clinical features alone.

A study of 12 *LRRK2-GBA* dual mutation carriers (all with *LRRK2* G2019S; 9 with *GBA* N370S, 2 with E326K and 1 with R496H) among 556 PD patients reports no significant differences in clinical motor scores, motor fluctuations, freezing of gait, and number of patients reaching Hoehn & Yahr stage 3 compared with carriers of single-mutation or non-carriers [93]. However, *GBA*-PD patients (N370S being the most common) show higher rates of dementia, RBD and psychosis while dual mutation carriers have the least RBD and psychosis, suggesting that *LRRK2* G2019S may exert a protective effect among patients with *GBA* mutations. In a larger study, 27 dual mutation patients (all with *LRRK2* G2019S and a majority with mild *GBA* mutations) have significantly better motor function, lower rates of dementia and slower cognitive decline than both mild and severe *GBA* mutation carriers, again implying a modifying role of *LRRK2* on motor and nonmotor phenotypes of patients with *GBA* mutations [94]. Combining data from multiple studies, Ortega and colleagues showed that *GBA-*PD (containing similar proportions of patients with mild and severe *GBA* mutation to the dual mutation group) have the fastest motor and cognitive decline compared with *LRRK2*-PD, PD with dual mutations and iPD, while the latter three groups are similar on this aspect [95]. Data concerning the age of onset of *LRRK2-GBA* dual mutation carriers are conflicting. Two studies reported that PD patients with *LRRK2-GBA* dual mutations were younger at first motor symptom onset than single mutation carriers [93, 96], while no such differences were found in two other studies [94, 95].

Collectively, these studies show that *LRRK2-GBA* dual mutation carriers have similar motor and non-motor symptoms to *LRRK2* carriers, which are milder than those seen in *GBA*-PD. Furthermore, dual mutation carriers have milder clinical features even when compared with *GBA*-PD patients carrying the mild *GBA* N370S mutation, suggesting that the consistently worst phenotype in *GBA*-PD is not driven by those carrying severe *GBA* mutations [94]. There are limitations to these studies: (1) the numbers of dual mutation carriers in most studies are small, and (2) most studies included only one *LRRK2* mutation (G2019S) and hence it is not clear if other *LRRK2* mutations have the same effect. Nevertheless, the observations from these studies challenge the notion of an additive deleterious effect of dual mutations suggested by (1) the findings of increased risk of PD and earlier age of onset in dual mutation carriers compared with single mutation carriers [93, 96] and (2) the finding in cell models of improved GCase activity after treatment with LRRK2 kinase inhibitor [12–14]. Furthermore, increased GCase activity has been found in dried blood spots of *LRRK2* G2019S PD patients, again suggesting a protective effect of LRRK2 leading to compensatory increase in GCase activity, although it is not known whether GCase activity in blood reflects its activity in the brain [97]. Clearly, the interaction between *LRRK2* and *GBA* is complex. Given that both *LRRK2* and *GBA* mutations have incomplete penetrance in PD, other unknown factors are likely to affect the overall risk and clinical progression of PD. Further studies are needed to clarify how the two genes interact to affect the phenotype and whether this interaction represents an opportunity for disease modification.

## Therapeutic strategies targeting *GBA *and *LRRK2*

The ALP is regulated by *LRRK2* and *GBA* along with several other PD-associated genes. Its disturbance is a key mechanism in the pathogenesis of PD (reviewed in [11]). Since there is strong evidence linking lysosomal dysfunction with α-syn aggregation and propagation, therapeutic strategies to enhance autophagy and improve lysosomal dysfunction are being employed in disease modification of PD. GBA is well studied for its role in maintaining normal lysosomal function. Its bi-directional relationship with α-syn metabolism suggests that enhancement of GCase activity will be beneficial not only in *GBA* mutation carriers but in iPD as well. Strategies to mitigate the effects of reduced GCase activity are mainly headed in two directions: (1) small molecule chaperones to facilitate transit of GCase to the lysosome and (2) substrate reduction therapy to inhibit biosynthesis of GlcCer (reviewed in [98]).

Ambroxol, which is widely used as a mucolytic agent, has been shown to increase GCase activity in fibroblasts from healthy controls, *GBA* carriers (with or without PD) and iPD patients [99, 100] with associated improvement in functional lysosomal mass and proteolytic activity. Transgenic mice overexpressing α-syn have reduced GCase activity compared with WT control mice, confirming that elevated α-syn can lead to reduced WT GCase activity [101]. Ambroxol treatment of these mice increases GCase activity and reduces α-syn and pS129-α-syn levels in brain. Another study employing a rat model of PD with unilateral intrastriatal injection of 6-hydroxydopamine (6-OHDA) shows that ambroxol treatment initiated 4 weeks after 6-OHDA injection (when motor symptoms have fully developed and nigral cell loss has reached maximal levels) results in restoration of GCase activity, restoration of the dopaminergic system measured by tyrosine hydroxylase and DA transporter levels, reduction in α-syn pathology, and recovery of behavioral symptoms [102], suggesting disease-modifying effects in PD. Ambroxol has also been tested in human subjects. A single-center, open-label noncontrolled clinical trial with *GBA*-PD and iPD patients (ClinicalTrials.gov Identifier NCT02941822) shows that ambroxol achieves good cerebrospinal fluid penetration and improves motor symptom scores [103]. A phase II placebo-controlled clinical trial (ClinicalTrials.gov Identifier NCT02914366) is currently recruiting PD patients with mild-to-moderate dementia to study the disease-modifying effects of ambroxol. Another approach to mitigate the effects of reduced GCase activity is to inhibit the synthesis of GlcCer. In mouse models of PD, treatment with a GlcCer synthase inhibitor has been shown to reduce GlcCer in the brain, slow the accumulation of hippocampal α-syn aggregates, and improve memory deficits [104]. Another GlcCer synthase inhibitor has been shown to reduce GlcCer levels in *GBA* mutant mouse brain and to rescue lysosomal deficits, reduce α-syn pathology and DA neuronal cell loss in mouse neurons [105]. In humans, Venglustat (an oral GlcCer synthase inhibitor) has been shown in a phase I study to be well tolerated and a phase II trial has recently completed recruitment (ClinicalTrials.gov Identifier NCT02906020) [106].

Increased kinase function in *LRRK2* mutations represents a toxic “gain-of-function” mechanism causing autophagic dysfunction, and is an attractive target for pharmacologic intervention. In cell and transgenic animal models overexpressing mutant *LRRK2*, LRRK2 kinase inhibition has been shown to reduce pS129-α-syn accumulation, oligomeric α-syn levels and α-syn release [14, 42, 43], and attenuate neurite shortening and DA neuronal death (reviewed in [107]). Going forward, mouse models with *LRRK2* knockin mutation incorporate genetic susceptibility and aging to model PD pathogenesis and can be very useful in the study of the *in vivo* effects of LRRK2 kinase inhibition [108, 109]. An important consideration of using LRRK2 inhibition as a treatment strategy of PD is its safety profile. Since *LRRK2* is expressed not only in the brain but also in kidney, lung and immune cells, long-term LRRK2 kinase inhibition could potentially affect these tissues. Mice and non-human primates do not exhibit any renal toxicity after receiving LRRK2 kinase inhibitor treatment [107, 110, 111]. In contrast, abnormal cytoplasmic accumulation of lysosome-related lamellar bodies in type II pneumocytes has been noted in the lungs of rodents and non-human primates after LRRK2 kinase inhibition [111, 112]. These abnormalities appear to be reversible on drug withdrawal and, more importantly, lower doses of LRRK2 kinase inhibitor which can achieve substantial brain LRRK2 kinase inhibition do not induce lung pathology [113], indicating a safety margin where brain LRRK2 kinase is inhibited without adverse effects on the lungs. It is of much interest to know whether LRRK2 kinase inhibition can be a viable treatment strategy beyond *LRRK2* mutation carriers. In particular, *LRRK2* and *GBA* mutations show substantial biological overlap in their effects on ALP impairment and α-syn pathology. LRRK2 reduces GCase activity by phosphorylating Rab10 [14]. In cell models, LRRK kinase inhibition has been shown to increase GCase activity and reduce pS129-α-syn levels in neurons carrying *LRRK2* or *GBA* mutations. In addition, variants in regions around the *LRRK2* locus have been identified in GWAS of sporadic PD patients [24]. Hence, it is conceivable that LRRK2 inhibition may be useful in *GBA*-PD and a subset of sporadic PD patients. One LRRK2 kinase inhibitor, DNL201, is in phase I clinical trial that has just completed recruitment of PD patients with and without *LRRK2* mutation (ClinicalTrials.gov Identifier NCT03710707).

Apart from directly modulating enzymatic activities of LRRK2 kinase and GCase, the general abnormalities in ALP as revealed in postmortem PD brain samples as well as cell and animal models suggest that modulation of pathways to enhance autophagy may also be viable therapeutic options. For example, farnesyltransferase inhibitors have been shown to enhance GCase activity, reduce α-syn aggregation and improve neuronal viability in PD patient-derived iPSC-midbrain neurons expressing A53T mutant α-syn by promoting hydrolase trafficking to the lysosome [11, 114]. Inhibition of mTOR promotes macroautophagy and ALP, and induces nuclear translocation of transcription factor B (TFEB), thus activating transcription of autophagic and lysosomal proteins [11, 115]. Hence, inhibitors of mTOR, such as rapamycin, represent another treatment strategy. Activation of CMA may improve α-syn degradation. Treatment of cells and mice with CMA activators has been shown to reduce α-syn accumulation and release [11, 50, 116]. Nilotinib, a tyrosine kinase inhibitor which activates autophagy through the AMPK pathway, has been shown to reduce α-syn levels, suppress DA neuronal loss and improve motor deficits in mice [117]. However, results in a human clinical trial recently published have been disappointing. Nilotinib achieved low CSF penetrance with no improvement in clinical motor scores in patients with moderately advanced PD [118]. Further advancements in our understanding of the regulation of ALP will hopefully lead to new therapeutic targets in the disease modification of PD.

## Conclusions and future directions

**Fig. 3:**
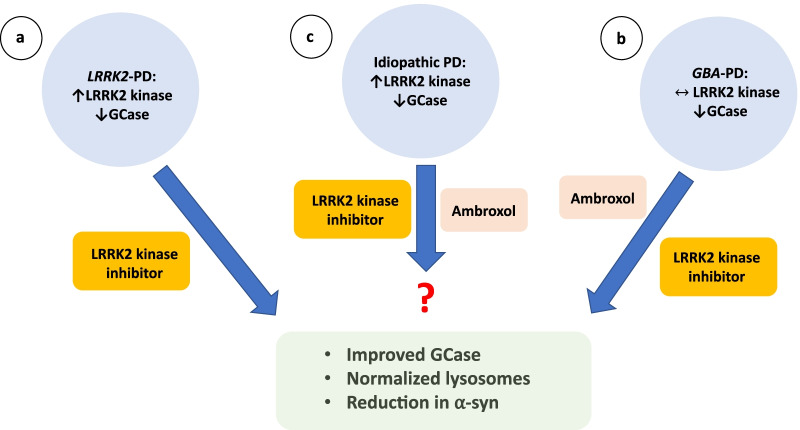
Treatment targeting LRRK2 kinase and GCase activities in PD. **a** In *LRRK2*-PD, LRRK2 kinase activity is increased and GCase activity is reduced. Treatment with LRRK2 kinase inhibitor has been shown to improve GCase activity, normalize lysosome function and reduce α-syn. **b** In *GBA*-PD, GCase activity is reduced while LRRK2 kinase activity is normal. Both ambroxol and LRRK2 kinase inhibitor have been shown to improve GCase activity, lysosomal function and reduce α-syn. **c** Idiopathic PD has been shown to have increased LRRK2 kinase and reduced GCase activities; it remains to be seen whether treatment with ambroxol or LRRK2 kinase inhibitor will be useful

The identification of *LRRK2* and *GBA* mutations in familial and sporadic PD has led to major advancement in the past 10 years in our understanding of the regulation of ALP. The lysosome has emerged to be a critical player in maintaining α-syn homeostasis and is also where the effects of *LRRK2* and *GBA* mutations converge. Impairment of lysosomal function causes broad abnormalities in autophagy that ultimately lead to accumulation of toxic oligomeric α-syn, which further impairs autophagy, forming a vicious cycle. Mitochondrial dysfunction and impaired mitophagy have also been described in *LRRK2* and *GBA* mutations, which are likely linked to reduced efficiency of ALP [119, 120]. Specifically, in a mouse model with heterozygous *GBA* L444P mutation and another mouse model with *LRRK2* R1441C homozygous knockin mutation, accumulation of mitochondria with abnormal morphology, increased oxidative stress, reduced ATP production, increased accumulation of autophagosomes with reduced rate of mitophagy has been described [119, 120]. These abnormalities are consistent with mitochondrial dysfunction observed in PD, with impaired electron transport chain function, impaired calcium buffering and abnormal mitochondrial morphology and dynamics (reviewed in [121, 122]). Furthermore, *LRRK2* and *GBA* have also been implicated in immune response, indicating their multi-faceted functions [123]. There are still huge gaps in our knowledge. It is unclear at present why LRRK2 kinase activity is increased in iPD or how α-syn impairs trafficking of GCase from ER to the lysosome. Furthermore, in the majority of PD patients who have no known mutations, it is unclear what triggers the pathogenic cascade leading to lysosomal dysfunction and α-syn accumulation. Nevertheless, altered LRRK2 and GCase activities and their associated autophagic defects have been observed in iPD, potentially extending the application of drugs that modulate their functions to the wider PD population (Fig. 3). For example, ambroxol and LRRK2 kinase inhibitors have both been shown to increase GCase activity in WT cells, and LRRK2 kinase inhibitors correct lysosomal defects in *GBA* mutant cells. Clinical trials of some of these drugs are underway and their results, particularly in iPD patients, will be eagerly awaited.

## Data Availability

Not applicable.

## Abbreviations

ALP: Autophagic-lysosomal pathway
AMPK: Adenosine monophosphate (AMP)-activated protein kinase
AV: Autophagic vacuoles
CMA: Chaperone-mediated autophagy
DA: Dopamine
ER: Endoplasmic reticulum
ERK: Extracellular signal regulated protein kinase
GBA: Glucocerebrosidase
GCase: Glucocerebrosidase
GD: Gaucher disease
GlcCer: Glucosylceramide
GWAS: Genome-wide association study
iPD: Idiopathic Parkinson’s disease
iPSC: Induced pluripotent stem cells
LAMP1: Lysosome-associated membrane protein 1
LAMP2A: Lysosome-associated membrane protein 2A
LB: Lewy bodies
LC3: Light chain 3
LIMP2: Lysosomal integral membrane protein 2
LRRK2: Leucine-rich repeat kinase 2
MEK: Mitogen activated protein kinase/extracellular signal regulated protein kinase (MAPK/ERK) kinase
mTOR: Mammalian target of rapamycin
NAADP: Nicotinic acid adenine dinucleotide phosphate
PD: Parkinson’s disease
RBD: Rapid eye movement sleep behavior disorder
